# Electrochemical Immunosensor Based on Nanoelectrode Ensembles for the Serological Analysis of IgG-type Tissue Transglutaminase

**DOI:** 10.3390/s19051233

**Published:** 2019-03-11

**Authors:** Henok B. Habtamu, Tarcisio Not, Luigina De Leo, Sara Longo, Ligia M. Moretto, Paolo Ugo

**Affiliations:** 1 Department of Molecular Sciences and Nanosystems, University Ca’Foscari of Venice, via Torino 155, 30172 Venezia Mestre, Italy; henbab12@yahoo.com (H.B.H.); sara.longo@yahoo.it (S.L.); moretto@unive.it (L.M.M.); 2 Institute for Maternal and Child Health - IRCCS “Burlo Garofolo”, 34100 Trieste, Italy; tarcisio.not@burlo.trieste.it (T.N.); deleo.luigina@libero.it (L.D.L.); 3 Department of Medical, Surgical and Health Sciences, University of Trieste, 34100 Trieste, Italy

**Keywords:** celiac disease, immunosensor, anti-tissue transglutaminase, nanoelectrode array, voltammetry

## Abstract

Celiac disease (CD) is a gluten-dependent autoimmune disorder affecting a significant percentage of the general population, with increasing incidence particularly for children. Reliable analytical methods suitable for the serological diagnosis of the disorder are urgently required for performing both the early diagnosis and the follow-up of a patient adhering to a gluten-free diet. Herein we report on the preparation and application of a novel electrochemical immunosensor based on the use of ensembles of gold nanoelectrodes (NEEs) for the detection of anti-tissue transglutaminase (anti-tTG), which is considered one reliable serological marker for CD. To this end, we take advantage of the composite nature of the nanostructured surface of membrane-templated NEEs by functionalizing the polycarbonate surface of the track-etched membrane with tissue transglutaminase. Incubation of the functionalized NEE in anti-tTG samples results in the capture of the anti-tTG antibody. Confirmation of the recognition event is achieved by incubating the NEE with a secondary antibody labelled with horseradish peroxidase (HRP): in the presence of H_2_O_2_ as substrate and hydroquinone as redox mediator, an electrocatalytic current is indeed generated whose increment is proportional to the amount of anti-tTG captured from the sample. The optimized sensor allows a detection limit of 1.8 ng mL^−1^, with satisfactory selectivity and reproducibility. Analysis of serum samples from 28 individuals, some healthy and some affected by CD, furnished analytical results comparable with those achieved by classical fluoroenzyme immunoassay (FEIA). We note that the NEE-based immunosensor developed here detects the IgG isotype of anti-tTG, while FEIA detects the IgA isotype, which is not a suitable diagnostic marker for IgA-deficient patients.

## 1. Introduction

Celiac disease (CD) is a gluten-dependent autoimmune disorder affecting approximately 1% of the general population [1,2]. The only treatment that reduces mortality and the development of CD-associated disorders is a gluten-free diet [3]. However, some patients can be asymptomatic for years, so in the absence of suitable diagnostic tools, the disease diagnosis can be dangerously postponed. Up to a few years ago, definitive diagnosis of CD was based on detecting histological changes in small intestinal mucosa after biopsy. However, recent guidelines by the European Society of Pediatric Gastroenterology advise against intestinal biopsy in symptomatic CD patients with high serum concentrations of anti-tissue transglutaminase (anti-tTG) antibodies (10 times the cut-off value) [4,5].

Currently, CD-related antibodies are investigated by using optical ELISA, fluoroenzyme immunoassay (FEIA), or indirect immunofluorescence assay [6,7]. Immunoglobulin A (IgA) is the isotype typically determined as the target analyte for serological CD screening, but IgA-deficient CD patients are not identified by IgA serology; in these patients, immunoglobulin G (IgG) anti-tTG antibodies are investigated [8]. However, several clinical studies have shown that IgG anti-tTG antibodies are not as sensitive and specific as the IgA anti-tTG antibodies, and the use of the indirect immunofluorescence assay to detect anti-endomysium antibodies (EMA) in daily practice is limited by subjective interpretations [9]. The cut-off value for IgG anti-tTG antibodies measurement is 7 U/mL. This evidence indicates that the development of an IgG-based diagnostic method with improved sensitivity and specificity is urgently required. Many trials have been conducted to develop anti-tTG electrochemical sensors using different approaches to improve the analytical performances of functionalized electrode surfaces [10,11,12,13,14,15,16]; however, up to now, no unique confirmatory serological electrochemical biosensor has been adopted for practical use. 

Recent studies [17] demonstrated that the analytical performances of bioelectrochemical sensors can be improved by miniaturization of the transduction element down to the nanometer scale; this results in a dramatic enhancement of diffusive mass fluxes to the nanoelectrode as well as a lowering of the double-layer charging current, with both factors contributing to dramatic improvements in detection limits [18,19,20,21,22,23,24,25,26,27,28,29]. Moreover, unlike with the use of an individual nanoelectrode, arrays of nanoelectrodes do not require high amplification of current signals nor the use of shielding devices such as a Faraday cage. Arrays indeed provide higher faradaic currents, along with significant improvements to the accuracy and precision [27,28,29,30,31]. Arrays of nanoelectrodes can be fabricated with an ordered spatial distribution or with a random distribution to obtain the so-called nanoelectrode ensembles (NEEs) [22,27,32,33,34]. 

**Scheme 1:**
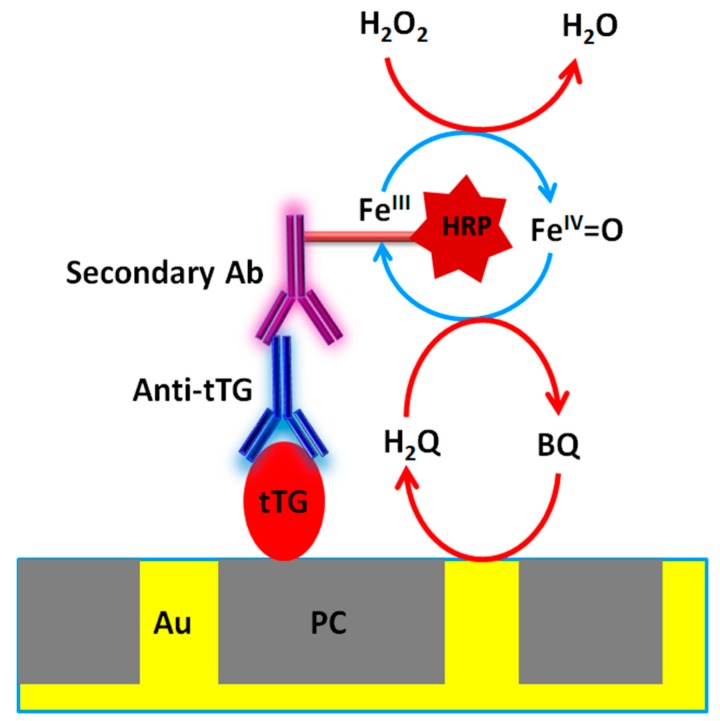
Scheme depicting the immunosensor architecture and the associated electrochemical and enzymatic processes, where PC= polycarbonate; tTG = tissue transglutaminase; Anti-tTG = antibody for tissue transglutaminase; HRP = horseradish peroxidase; H_2_Q = hydroquinone; BQ = benzoquinone. Components are not drawn to scale; the average diameter of the Au nanoelectrodes is 30 nm and their average center-to-center distance is 200 nm.

In this work, we employ NEEs prepared via template electroless deposition of gold within the pores of polycarbonate (PC) track-etched membranes [22,32]. When the boundaries of the radial diffusion layers of each nanoelectrode overlap, the so-called total overlap (TO) diffusion regime is achieved [22,34]. NEEs prepared from commercially available track-etched PC membranes (typically with an average pore diameter of 30 nm, average pore distance of 200 nm, and pore density of 6 × 10^8^ pores/cm^2^) operate under the TO regime, providing peak-shaped voltammetric signals. The faradaic peak current is proportional to the geometric area (*A*_geom_, that is, the area of the nanoelectrodes and the insulator between them exposed to the electrolyte), while the double-layer charging current depends only on the active area (*A*_act_, i.e., the area of the metal nanoelectrodes only). Typically, for NEEs, the *A*_geom_/*A*_act_ ratio (that is, the reciprocal of the fractional area, *f*) is of the order of 10^2^–10^3^, allowing one to achieve detection limits 2–3 orders of magnitude lower than those obtained with millimeter-sized electrodes with the same geometric area [22,33]. For biosensing purposes, an additional advantage of NEEs is the possibility of binding biomacromolecules (proteins or DNA) onto the PC membrane used to prepare the nanoelectrode ensemble [27]. The surface of the PC membrane contains a significant surface concentration of pendant carboxylic groups, which can interact with the amino terminations of the biomolecules [35,36]. Several biosensors have been recently developed using this strategy for detecting DNA [37], proteins [38,39,40], or enzyme substrates [41]. In the present work, we take advantage of the potentialities offered by NEEs by developing an anti-tTG electrochemical sensor, the architecture of which is summarized in Scheme 1. 

The biorecogniton element is composed of molecules of the tTG antigen adsorbed onto the PC which surrounds the nanoelectrodes. It captures the target analyte, namely, anti-tTG, which is then reacted with a secondary antibody labelled with HRP. Finally, the binding of HRP is detected by adding H_2_O_2_ as substrate and hydroquinone (H_2_Q) as redox mediator, thus modulating, via the electrochemical reduction of benzoquinone (BQ), the enzymatic reduction of H_2_O_2_ by HRP. It is worth noting that our group recently applied a similar NEE-based architecture in the development of a novel immunosensor for the detection of anti-tTG [42] which employs electrochemiluminescence (ECL) as the detection technique. ECL indeed allows us to achieve high sensitivity and very low detection limits (for anti-tTG as low as 470 pg/mL [42]); however, it requires the use of a quite complex sensor architecture in which a biotinilated secondary Ab is bound to a streptavidin-modified ruthenium label, generating ECL by reaction with a co-reactant, namely, tri-propylamine [42]. In the electrochemical (EC) sensor studied here, the use of voltammetry as the detection technique allowed us to exploit a simpler and more conventional sensor architecture based on the use of HRP-labeled goat anti-human IgG as the secondary antibody (see Scheme 1). In the present work, after detailed characterization of the sensor and optimization of its analytical performances, special focus is put in demonstrating its possible use for diagnostics purposes in human serum samples by analyzing anti-tTG in serum samples from 28 pediatric patients and comparing the sensors’ responses with results obtained using existing analytical methods. 

## 2. Experiment Details

### 2.1. Instrumentation

Cyclic voltammetry (CV) was performed with a CHI 660B potentiostat using a three-electrode electrochemical cell where the NEE was the working electrode, a Pt spiral was the counter electrode, and Ag/AgCl, KCl saturated was the reference electrode.

Scanning electron microscopy (SEM) and energy-dispersive X-ray spectroscopy (EDS) analysis were performed using a TM3000 Hitachi tabletop scanning electron microscope coupled with a SwiftED3000 X-Ray microanalysis system.

### 2.2. Chemicals and Materials

Gold electroless plating solution (Oromerse Part B, Technic Inc., Cranston, RI, USA), bovine serum albumin (BSA), Tween 20, hydrogen peroxide 30%, and hydroquinone (H_2_Q) 99% were purchased from Sigma. Buffer solutions, carbonate–bicarbonate buffer pH 9.2, 0.01 M phosphate buffer saline (PBS) pH 7.4, and 0.05 M phosphate buffer (PB) pH 7.4 were prepared with distilled water. All other reagents were of analytical grade.

Human recombinant tissue transglutaminase (h-tTG) was prepared as previously described [1]. Monoclonal mouse anti-human tTG antibody (CUB 7402) was purchased from BioOptica, HRP-labeled goat anti-human IgG (whole molecule) from Sigma-Aldrich, and HRP-labeled goat anti-mouse pAb IgG secondary antibody from Abcam. All serum samples were from pediatric patients aged between 2 and 16 years and were supplied by Burlo Garofolo Pediatric Institute (Trieste, Italy); the ethical committee assurance number is CE/V-131. All the biologicals including BSA and Tween 20 were diluted using 0.01 M PBS pH 7.4. 

Track-etched PC filter membrane (47 mm overall filter diameter, 6 µm thickness, impregnated by the producer with polyvinylpyrrolidone) with an average pore diameter of 30 nm and pore density of 600 million pores per cm^2^ from SPI supplies (West Chester, PA, USA) was used as a template for the preparation of the nanoelectrode ensembles.

### 2.3. Methods

#### 2.3.1. Fabrication of the NEEs

NEEs were prepared by electroless deposition of gold in track-etched PC filter membranes using an already published procedure [22] and its following updates [32,36]. During the electroless deposition of gold from a solution, each pore of the PC membrane was filled with a metal nanowire whose head was exposed to the electrolyte solution so that the surface of the NEE was composed of millions of randomly spaced gold nanodisks. The membrane bearing the nanodisks was assembled into electrodes of suitable geometry and size as previously described [34]. Typical values for *A*_geom_ and *A*_act_ for the NEEs used in this work were 7 × 10^−2^ cm^2^ and 6 × 10^−4^ cm^2^, respectively.

#### 2.3.2. Immunosensor Construction and Electrochemical Detection

An aliquot of 10 µL of 10 µg mL^−1^ tTG solution in 0.1 M carbonate buffer, pH 9.2, was dropped on the NEE and incubated for 2 h at 25 °C to obtain what we will call here TGNEE. It has been previously shown that under these conditions, antigen and antibody proteins bind preferentially on the PC of NEEs [38,39,40,41,42]. The TGNEE was subsequently blocked with 1% BSA in 0.01 M PBS, pH 7.4, for 30 min, followed by incubation for 60 min with 10 µL standard solution of anti-tTG or serum sample, diluted 1:200 with 0.01 M PBS, pH 7.4 (unless differently indicated). After washing with 0.01 M PBS, pH 7.4, containing 0.05% Tween 20, the captured primary antibody was coupled with 20 µL of 10 µg mL^−1^ HRP-labeled goat anti-mouse IgG secondary antibody in 0.01 M PBS, pH 7.4, for 60 min. Incubations were carried out at room temperature (22 ± 1 °C) in a water-vapor-saturated vessel. All washing procedures were followed by gentle drying with N_2_ flow. Finally, the immunosensor was dipped in an electrochemical cell containing freshly prepared 1 mM H_2_Q in 0.05 M phosphate buffer (pH 7.4). The H_2_Q solution was prepared under N_2_ in an amber glass bottle to avoid oxidation by atmospheric O_2_ and light. All electrochemical measurements were performed at room temperature in an electrolyte solution deoxygenated with N_2_. Cyclic voltammograms were recorded before and after addition of 1.5 mM H_2_O_2_ at scan rate of 50 mV s^−1^ between −0.65 and +0.85 V. Negative controls were performed in the absence of anti-tTG. 

Fluoroenzyme immunoassay for anti-tissue transglutaminase antibodies was performed according to the manufacturer’s instructions (Elia^TM^ Celikey, Phadia 250).

## 3. Results and Discussion

**Scheme 2:**
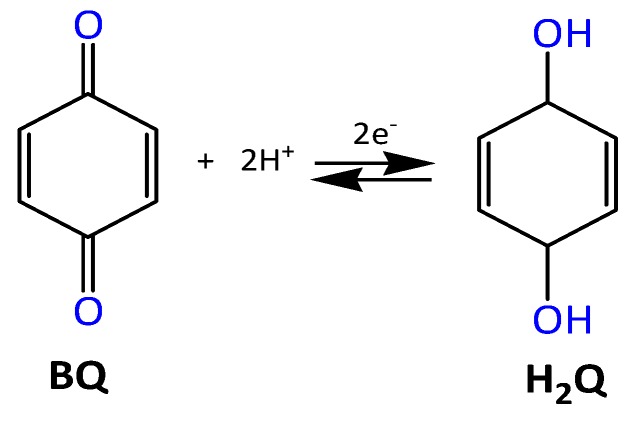
Electrochemical reduction of benzoquinone (BQ) to hydroquinone (H_2_Q).

**Figure 1:**
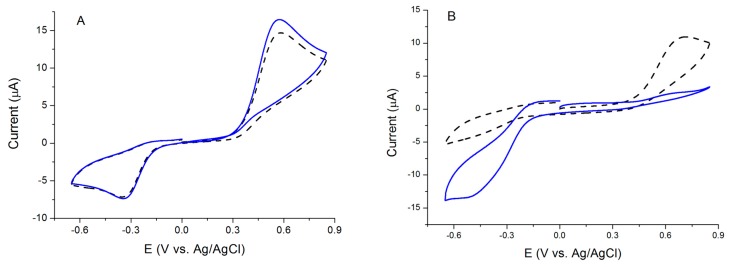
Cyclic voltammograms recorded at 50 mV s^−1^ in 1 mM H_2_Q, 0.05 M PBS (pH 7.4) (**A**) with a bare nanoelectrode ensemble (NEE) before (dashed line) and after (solid line) the addition of 1.5 mM H_2_O_2_; and (**B**) with a NEE functionalized with transglutaminase incubated with anti-tTG and then with secAb-HRP (i.e., TGNEE–antiTG–secAb-HRP), before (dashed line) and after (solid line) the addition of 1.5 mM H_2_O_2._

Figure 1A (dashed line) shows the cyclic voltammogram recorded with a bare NEE in 1 mM H_2_Q, 0.5 M PBS (pH 7.4). It is characterized by a reduction peak at −0.27 V to which an oxidation peak at 0.64 V is associated, with both peak currents scaling linearly with the H_2_Q concentration (in the range 1–5 mM) and the square root of the scan rate (in the range 20–200 mV s^−1^, not shown). The full line CV in Figure 1A shows that the addition of 1.5 mM H_2_O_2_ does not cause any significant change in the voltammetric pattern, thus excluding the occurrence of any direct reaction between H_2_Q and H_2_O_2_ in these experimental conditions. These results agree with the previous literature for macroelectrodes [43,44,45,46,47,48,49,50,51] which attributed the observed CV signals to the 2-electrons/2-protons electrochemical oxidation of H_2_Q to BQ and following re-reduction, according to reaction (1) and Scheme 2:

H_2_Q → BQ + 2 e + 2 H^+^ (1)

Indeed, the forward-to-backward peak separation observed with the NEE is quite large (approximately 0.9 V), aligning more with the value observed for H_2_Q electrochemistry at chemically modified macroelectrodes [44,50] than with the much smaller value observed with bare macroelectrodes (0.1 V). Note that, in any case, the voltammetric behavior of the H_2_Q/BQ is not perfectly reversible. The large peak-to-peak separation observed with the NEE agrees with the fact that these electrode arrays behave as electrodes with a partially blocked surface (PBSEs) [22,33,52,53,54,55,56], where the nanodisk electrodes of the NEEs correspond to the unblocked surfaces of the PBSE, while the PC template is the blocking material [22]. Therefore, the kinetics of heterogeneous electron transfer are apparently slowed down by a factor that depends on fractional area, such that redox systems which are quasi-reversible in nature (e.g., H_2_Q) behave as electrochemically irreversible systems at NEEs [22,33]. 

Figure 1B reports the CV patterns recorded for 1 mM H_2_Q with a TGNEE, incubated at first with anti-tTG and then with secAb-HRP (see Section 2 for details), from here on named tTGNEE–anti-tTG–secAb-HRP. The dashed and full line CVs refer to voltammograms recorded in the absence and presence of 1.5 mM H_2_O_2_, respectively.

The former CV pattern (Figure 1B, dashed line) is similar with the one recorded with a bare NEE (Figure 1A, dashed line), indicating that the functionalization of the NEE does not interfere with the electron transfer.

On the contrary, the full line CV in Figure 1B shows that a dramatic change is detected with the TGNEE–antiTG–secAb-HRP electrode system when 1.5 mM H_2_O_2_ is added to the electrolyte solution. A sharp increase of the reduction current with a slight shift in peak potential to −0.35 V is detected, the peak tending to acquire a partially sigmoidal profile. Note that, concomitantly, the re-oxidation peak at +0.64 V disappears. 

The above experimental evidence supports the hypothesis of the occurrence at the TGNEE–antiTG–secAb-HRP sensor of the following enzyme-mediated electrocatalytic cycle:

BQ + 2 e + 2 H^+^ → H_2_Q (2)

H_2_O_2_ + HRP (Fe^3+^) → HRP(Fe^4+^ = O) + H_2_O (3)

HRP(Fe^4+^ = O) + H_2_Q → HRP(Fe^3+^) + H_2_O + BQ. (4)

Reaction (3) between the oxidized HRP label and H_2_Q agrees with the observed increase of the cathodic peak current associated with Reaction (1) as well as with the disappearance of the oxidation peak at +0.7 V, since H_2_Q is continuously reoxidized chemically rather than electrochemically.

All this evidence confirms the efficiency of the biorecognition–detection approach described in Scheme 1.

### 3.1. Optimization of Signal Detection Parameters

To optimize the immunosensor response, the role of the concentration of the enzyme substrate H_2_O_2_ and the mediator H_2_Q was studied while keeping the concentration of the anti-tTG in the incubation bath constant at 1 µg mL^−1^.

**Figure 2:**
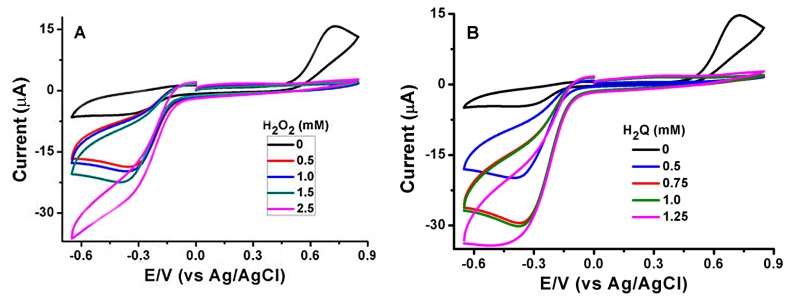
Cyclic voltammograms recorded at 50 mV s^−1^ with deoxygenated 0.05 M PBS (pH 7.4) containing (**A**) 1 mM H_2_Q and 0.0, 0.5, 1.0, 1.5, 2.5 mM H_2_O_2_; or (**B**) 1.5 mM H_2_O_2_ and 1.0, 1.5, 1.75, 2.0, 2.25 mM H_2_Q.

Figure 2A shows the changes in the CVs obtained with the TGNEE–AntiTG–secAb-HRP for H_2_O_2_ concentrations in the range 0–2.5 mM at constant H_2_Q concentration (namely, 1.0 mM). The electrocatalytic current shows a slight increase with the hydrogen peroxide concentration up to 1.5 mM. For H_2_O_2_ concentrations of >1.5 mM, the correct evaluation of *I*_cat_ was hampered by the rise in a sloping background current which is probably related to the electrochemical reduction of excess H_2_O_2_ [57,58]. Figure 2B reports the effect of the concentration of the redox mediator (at constant H_2_O_2_) on the cyclic voltammograms. The cathodic peak current increases with the concentration of H_2_Q up to 1 mM, levelling off for higher mediator concentrations and showing a saturation trend typical for mediated electrochemical processes. Based on these results, 1.5 mM H_2_O_2_ and 1.0 mM H_2_Q were chosen as the most suitable substrate and mediator concentrations for continuing the study.

**Figure 3:**
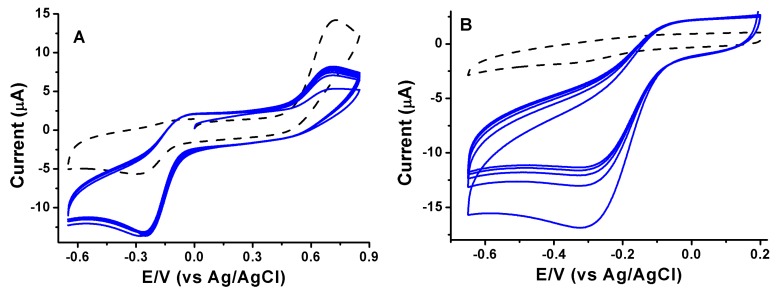
Continuous scan CVs recorded at 50 mV s^−1^ with the tTGNEE–Anti-tTG–secAb-HRP in 1 mM H_2_Q, 0.05 M PBS (pH 7.4) before (dash line) and after (solid lines) addition of 1.5 mM H_2_O_2_: (**A**) in the potential window −0.65 to 0.90 V, initial potential 0.0 V, initial scan direction: positive; (**B**) in the potential window −0.65 to 0.20 V, initial potential 0.20 V, initial scan direction: negative.

The effects of the scanning potential window and direction of the sweep on the CV responses of the immunosensor were also studied. Since the signal detection strategy in the immunosensor is based on the effect of the enzymatic Reaction (3) on the electrochemical reduction of BQ, we focused on the role of the width and initial direction of the voltammetric scan, which we performed in two alternative ways: (A) in the potential window −0.65 to 0.90 V, initial potential 0.0 V, and initial scan direction: positive; (B) in the potential window −0.65 to 0.20 V, initial potential 0.20 V, initial scan direction: negative. Typical voltammograms recorded under these conditions with the TGNEE–antiTG–secAb-HRP sensor, with and without H_2_O_2_ in the electrolyte, are illustrated in Figure 3A,B.

**Figure 4:**
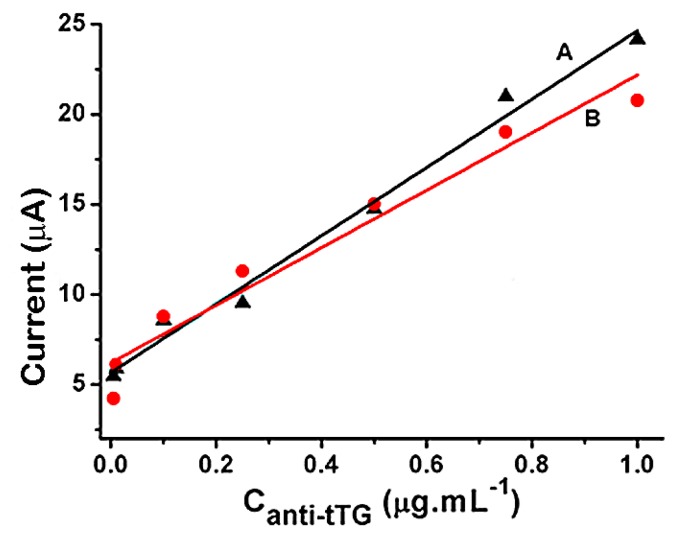
Calibration curves for the current recorded at 0.32 V vs. anti tTG concentration, obtained with the TGNEE–Anti-tTG–secAb-HRP immunosensor using approach (**A**) with the CV recorded in the potential window of Figure 3A or approach (**B**) with the CV recorded in the potential window of Figure 3B.

Figure 4 reports the calibration plots for the two approaches, obtained by incubating the TGNEE in tTG solutions prepared by serial dilutions of anti-tTG standards (Figure 3), followed by incubation with 20 µL of 10 µg mL^−1^ secAb-HRP.

Approach (A) was preferred since it provides better sensitivity (see the slopes of the calibration plots in Figure 4) and better reproducibility on repeated voltammetric scans (see Figure 3). Moreover, the possibility to observe the decrease or disappearance of the anodic peak related to Reaction (1) offers a direct experimental proof of the occurrence of the enzymatic process, thus providing qualitative evidence of a positive response in the case of a successful biorecognition event. Therefore, the potential window applied in all the following measurements was the one defined in approach A.

It can be pointed out that experiments performed in the absence of anti-tTG indicate a lack of electrocatalytic signal in the CVs recorded under such conditions. These tests gave similar results when performed in serum samples of healthy individuals (see below for details on the procedure), thus confirming the efficiency of the blocking with 1% BSA in avoiding undesired nonspecific binding as false positives. 

### 3.2. Analytical Performances

**Figure 5:**
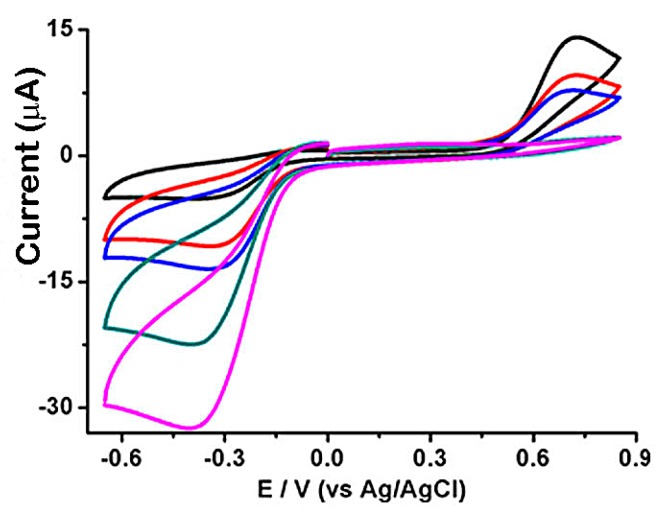
Cyclic voltammograms recorded with the TGNEE incubated with different concentrations of anti-tTG (0, 0.005, 0.01, 0.1, and 1 µg mL^−1^); other conditions as in Figure 1A.

The dependence of the detected catalytic peak current signal at the immunosensor on the anti-tTG concentration was studied over a wide antibody concentration range (0.001 to 10 µg mL^−1^). The experiments were performed in triplicate by incubating different TGNEEs with serial dilutions of monoclonal standard mouse anti-tTG. The CV responses of four representative concentrations are reported in Figure 5. 

**Figure 6:**
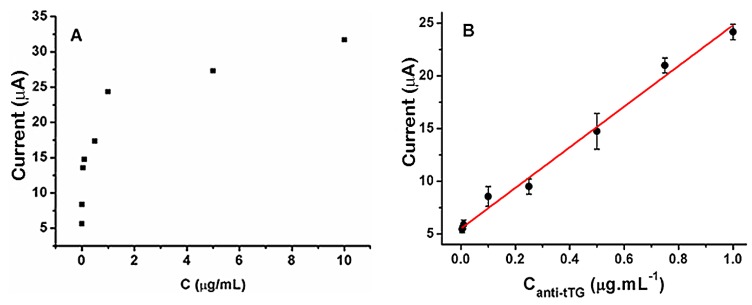
Dependence of the voltammetric current at −0.35 V on the anti-tTG concentration: (**A**) 0.00–10 µg mL^−1^; (**B**) 0.005–1.0 µg mL^−1^. Error bars indicate standard deviations of triplicate measurements (*n* = 3).

As shown in Figure 6A, the *I* values increase with the concentration of the anti-tTG (*C*_anti-tTG_), tending to an asymptotic profile for concentrations greater than 1 µg mL^−1^; however, a linear trend (*R*^2^ = 0.990) can be identified for concentrations of ≤1.0 µg mL^−1^ (see Figure 5B).

The precision (reproducibility) of the method was evaluated as the relative standard deviation (RSD) of repeated measurements (*n* = 10) performed with 0.5 µg mL^−1^ anti-tTG; the result was 2.6%. The detection limit (DL) was evaluated using the S/N = 3 criterion. In particular, the DL was calculated as DL = 3(SD/m), where SD is the standard deviation of the *y*-axis intercept and “m” is the slope (sensitivity) of the regression line, giving a result of 1.8 ng mL^−1^. The low detection limit, the wide linear range, and excellent reproducibility indicate that the TGNEE immunosensor offers analytical performances that are competitive with those achievable by other electroanalytical sensors developed for the same goals [11,12,13,14]. On the other hand, we wish to point out that the ECL sensor developed recently is characterized by a slightly lower detection limit (0.47 ng mL^−1^ vs. 1.8 ng mL^−1^), wider linear range, and higher sensitivity; however, it has the disadvantage of relying on a more complex analytical procedure and the use of more sophisticated and expensive equipment [42].

Trials of regenerating the NEEs from already-used immunosensors furnished unsatisfactory results; therefore, each analyte solution was analyzed using a different TGNEE. In order to evaluate the reproducibility between different NE-based sensors, the anti-tTG level in a standard analyte solution was determined using three different NEE immunosensors. The standard deviation of this inter-assay (*n* = 3) was 7% when using 1 µg mL^−1^ anti-tTG as the reference analyte concentration. 

In order to study the stability of the immunosensor in storage, repeated determinations of 1 µg mL^−1^ anti-tTG were performed over a period of 7 days. The storage stability of the immunosensor was investigated by measuring the immunosensors’ catalytic current response every other day. When the immunosensor was not in use, it was stored in 0.01 M PBS, pH 7.4, at 4 °C. The observed loss in sensitivity was 5% on Day 3 and 25% on Day 7 after the preparation of the sensor. These results suggest that the immunosensor can be stored for only a few days after the immobilization of tTG. Anyhow, preparing fresh TGNEEs is not a main problem since it requires the simple incubation of an NEE (or a set of NEEs) in 0.1 M carbonate buffer, pH 9.2, containing tTG (10 µg mL^−1^), as described in Section 2.

### 3.3. Analysis of Clinical Serum Samples

The feasibility of the proposed immunosensor for clinical applications was evaluated by analyzing the anti-tTG IgG level of 28 clinical serum samples from pediatric patients aged between 2 and 16 years. Out of the 28 serum samples, 23 were taken from patients with a confirmed CD diagnosis, whereas the other 5 were taken from healthy individuals. The anti-tTG IgA levels of the CD positive samples ranged from 16 to 776 U mL^−1^ (see Table 1, as determined by FEIA). 

In order to set the optimum dilution of the serum samples, preliminary investigations were performed at 1:10, 1:50, 1:100, and 1:200 dilution ratios with 0.01 M PBS, pH 7.4. The 1:200 dilution was chosen for the analysis of all the serum samples due to its better catalytic peak current signal. It is worth noting that the dilution minimizes possible interference from other proteins present in the matrix [14].

**Figure 7:**
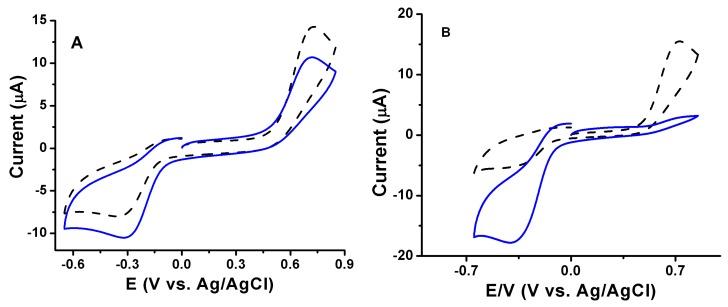
Cyclic voltammetric responses of serum samples recorded at 50 mV s^−1^ with the proposed immunosensor in 0.05 M PBS (pH 7.4) containing freshly prepared 1 mM H_2_Q before (dashed lines) and after (solid lines) addition of 1.5 mM H_2_O_2_: (**A**) serum sample from healthy subject (sample 10691), (**B**) serum sample from celiac patient (sample 10636).

Figure 7 compares the different signals recorded when analyzing the serum of non-celiac (Figure 7A) vs. celiac patients (Figure 7B), where the dashed and full line CVs were recorded in the absence and presence of H_2_O_2_, respectively. The latter CVs indicate that a relevant catalytic current increase is observed for the celiac patient’s sample, while a significantly smaller, almost negligible effect is observed for the healthy subject’s one. Quantification of signals based on the increase in the catalytic peak current was performed by interpolation with the calibration plot reported in Figure 6. 

Table 1 lists the final concentrations in serum of anti-tTG IgG determined by the electrochemical immunosensor (EC-IS) for all the 28 serum samples analyzed, taking into account the 1:200 dilution factor. They show that IgG data, measured by EC-IS, follow a trend parallel to that of the IgA values obtained by FEIA; however, the IgG concentrations are one order of magnitude smaller than the IgA concentrations. 

**Figure 8:**
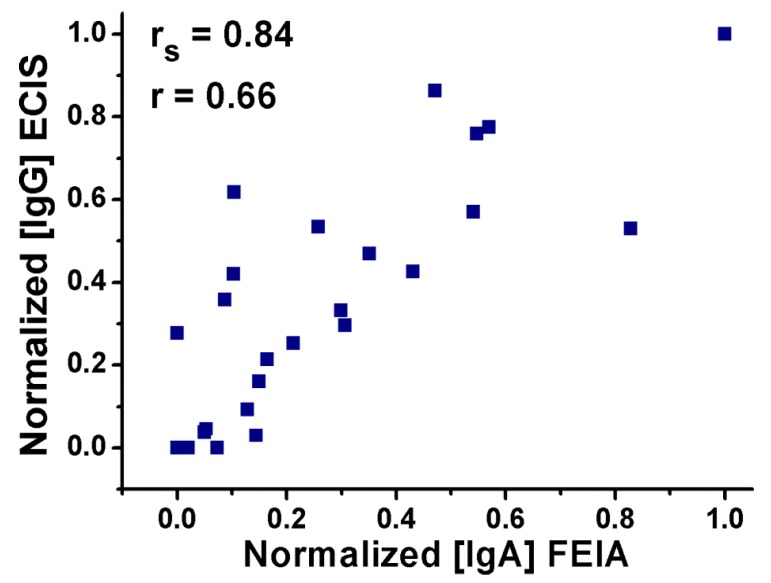
Scattered plot of normalized concentrations determined in real clinical serum samples obtained by the proposed electrochemical immunosensor (EC-IS) vs. the standard fluoroenzyme immunoassay (FEIA). Spearman’s coefficient (r_s_) of 0.84 and Pearson’s correlation (r) of 0.66 were obtained. Both data sets are normalized with respect to the higher concentration measured.

In order to evaluate the correlation quantitatively, statistical treatment was performed and the Spearman’s (r_s_) and Pearson’s (r) coefficients were calculated. The relevant plot is shown in Figure 8. 

The Spearman’s correlation (r_s_) is an important statistical parameter that measures the strength of a monotonic relationship between paired data sets [59]. The Pearson’s coefficient (r), on the other hand, quantifies the reliability of the existence of a linear relationship between the two data sets. In the case of satisfactory correlation, the values of the two correlation coefficients should range between 1 and −1.

From our results, the calculated the Spearman’s correlation coefficient to be 0.84 (>0.8), indicating a very strong correlation between the two data sets. The Pearson coefficient is 0.66, showing a strong positive linear relationship between the results of the two methods.

Besides the strong agreement between the IgG level measured using the new method and the corresponding IgA level determined by the clinical FEIA method, a preliminary set of experiments was conducted on a few serum samples to compare IgG data measured using our electrochemical immunosensor and that using FEIA. To this end, the IgG activity was measured in the same samples both by FEIA and by EC-IS, analyzing, in particular, some of the samples listed in Table 1 as well as four new samples. Since we were measuring the same antibody, namely, the IgG isotype of anti-tTG, we expressed the analyte concentration in terms of U/mL for both methods. The µg/mL values determined by EC-IS were indeed transformed into U/mL by using the value 5.5 as a conversion factor, calculated by measuring Inet changes for standard samples with known activity (in U/mL). The relevant results are reported in Table 2.

**Figure 9:**
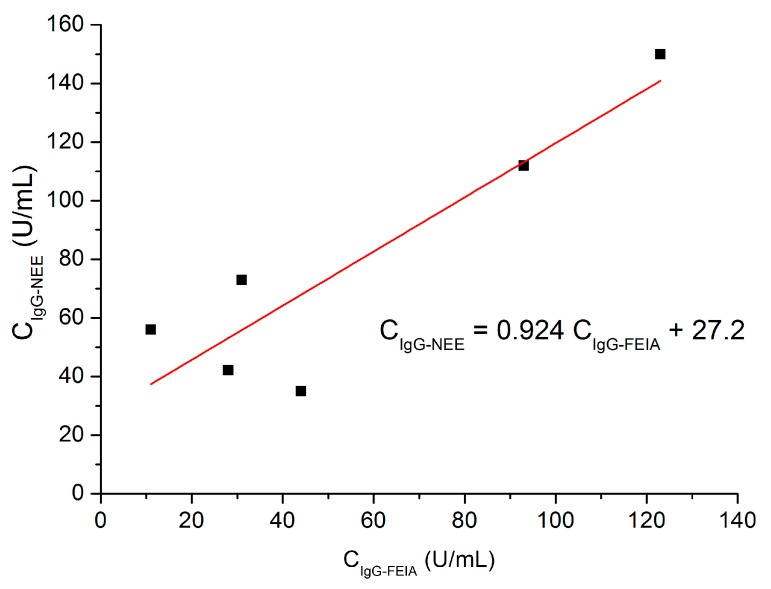
Correlation plot between anti-tTG IgG activities measured by the electrochemical immunosensor (C_IgG-NEE_) vs. FEIA analysis (C_IgG-FEIA_) in human serum samples.

Figure 9 shows the plot correlating the two data sets. Overall, a quite satisfactory agreement in the general trend is observed, with slope of 0.92; however, a significant scattering between the data can be observed, particularly at concentrations around 50 U/mL or lower. This evidence indicates that the EC-IS method is promising; however, further studies are required to improve its accuracy at small anti-tTG–IgG concentrations, as well as to set a new cut-off value based on a wider statistical basis.

## 4. Conclusions

A highly sensitive NEE-based electrochemical immunosensor was developed and evaluated for the determination of the IgG anti-tTG isotype involved in celiac disease. This is the first report demonstrating the application of arrays of nanoelectrodes as a platform for the purely electrochemical detection of CD biomarkers. The use of a smart biomolecule immobilization approach, where the capture antigen is selectively immobilized on the PC component of the Au NEEs, helped us to retain the desirable advantages of nanoelectrode ensembles, such as high S/N ratio. Moreover, the absence of any surface pretreatment step of the transducer for biorecognition element immobilization reduces the analytical steps and the reagents required, which is advantageous for transforming the proposed protocol to practical applications. 

The results presented in this work confirm the applicability of NEEs for the reliable detection of a disease biomarker, with a low detection limit of 1.8 ng mL^−1^. In the new biosensor design, the use of a redox mediator in the electrolyte solution is mandatory for achieving a high level of sensitivity. We note that a large proportion of the reporter label is relatively far (approximately up to 200 nm) from the surface of the nanodisk electrodes where the electrochemical signal is generated; this notwithstanding, they are reached by the mediator since NEEs operate under a total overlap diffusion regime, with the thickness of the diffusion layer in the time frame of the experiment being smaller than the hemi-distance between the nanoelectrodes. The immunosensor is able to detect the disease biomarker with a wide calibration linear range (0.005 to 1 µg mL^−1^) and with excellent signal reproducibility. 

In addition, the presented immunosensor is capable of detecting the anti-tTG IgG biomarker in clinical pediatric serum samples, offering strong agreement with the clinical outcome obtained for the IgA isotype using the FEIA method. Preliminary experiments conducted on a few serum samples showed that the new immunosensor furnishes results in basic agreement with those of the clinical FEIA method when the IgG isotype is measured by both methods, though the accuracy of the electrochemical immunosensor needs to be improved, particularly at low antibody concentrations. Further studies on larger data sets will be required in order to standardize the method, to evaluate its clinical sensitivity and specificity, and to set an anti-tTG IgG cut-off point for the EC-IS suitable to reliably discriminate between positive and negative serum samples. We note that further studies concerning the selectivity of the EC-IS for anti-tTG IgG vs. anti-tTG IgA types as well as on the possibility to electrochemically distinguish the two isotypes of this antibody are presently in progress [60].

## Figures and Tables

**Table 1:** Average anti-tTG IgG and IgA concentrations determined by the proposed electrochemical immunosensor (EC-IS) and clinical fluoroenzyme immunoassay (FEIA) methods, respectively.

Serum Sample	Anti-tTG IgG (µg/mL) by EC-IS	Anti-tTG IgA (U/mL) by FEIA	Serum Sample	Anti-tTG IgG (µg/mL) by EC-IS	Anti-tTG IgA (U/mL) by FEIA
10,669	41.3	366	10,644 *	13.22	0.1
10,658	15.855	232	10,550	27.28	420
10,636	37.08	442	10,526	36.34	425
10,668	1.405	112	10,525	0	16
10,637	17.12	68	10,521	2.15	41
10,650	22.4	273	10,519	1.75	39
10,623	12.06	165	10,633	47.88	776
10,607	0	57	10,645 *	0	0
10,603	14.14	238	10,691 *	0	0
10,605	7.68	116	9830	10.22	128
10,562	29.58	81	9777	25.54	200
10,577	20.06	80	9597 *	0	0
10,594	25.3	643	9463 *	0	0
10,554	20.38	334	9784	4.42	100

* Samples where clinical reports indicated absence of CD (healthy).

**Table 2:** Anti-tTG IgG activities measured by the proposed electrochemical immunosensor (EC-IS) and clinical fluoroenzyme immunoassay (FEIA) in human serum samples.

Serum Sample	Anti-tTG IgG (U/mL) by EC-IS	Anti-tTG IgG (U/mL) by FEIA
10,801	35	44
10,832	73	31
10,858	56	11
10,823	112	93
10,550	150	123
10,605	42	28
